# Solving coiled-coil protein structures

**DOI:** 10.1107/S2052252515003486

**Published:** 2015-02-26

**Authors:** Zbigniew Dauter

**Affiliations:** a Macromolecular Crystallography Laboratory, National Cancer Institute, Argonne National Laboratory, Argonne, IL 60439, USA

**Keywords:** molecular replacement, *ab initio* modeling, coiled-coil proteins

## Abstract

The successful approach to solving crystal structures of coiled-coil proteins with the program *AMPLE* is discussed.

With the availability of more than 100 000 entries stored in the Protein Data Bank (PDB) that can be used as search models, molecular replacement (MR) is currently the most popular method of solving crystal structures of macromolecules. Significant methodological efforts have been directed in recent years towards making this approach more powerful and practical. This resulted in the creation of several computer programs, highly automated and user friendly, that are able to successfully solve many structures even by researchers who, although interested in structures of biomolecules, are not very experienced in crystallography.

Several obstacles may make the process of MR difficult. Obviously, the search model has to be similar enough to the unknown structure to assure that the Patterson synthesis calculated from the model will resemble the analogous synthesis obtained from the diffraction data, since this is the basics of the MR approach. If potentially available search models are not sufficiently similar, it may be possible to rationally optimize them on the basis of the known sequence of residues, amino acids or nucleotides with programs dedicated to such tasks, such as *ROSETTA* (Shortle *et al.*, 1998 ▶), *QUARK* (Xu & Zhang, 2012 ▶), or *I-TASSER* (Lee & Skolnick, 2007 ▶). Sometimes it may be beneficial to use an ensemble of slightly different search models rather than a single one.

One of the most difficult problems hampering the process of MR is the presence in the investigated crystal structures of additional, non-crystallographic symmetry. This may be the result of the presence of several identical molecules arranged in a parallel fashion in the asymmetric part of the unit cell, or of internal symmetry of the individual molecule. An example of the latter situation is the case of coiled-coil proteins, which are built from a number of long α-helices wound around each other, forming a supercoil with long fragments that are parallel within one molecule and between neighboring molecules in the crystal. Such an architecture creates many identical self- and cross-Patterson vectors, highly confusing the process of MR. The coiled-coil proteins are, however, very important for a large number of biological processes, such as transmembrane signaling and transport, transcription, and many others.

Two publications in the current issues of **IUCrJ** and *Acta Cryst. D* address this important methodological problem. The paper by Thomas *et al.* (2015 ▶) in **IUCrJ** presents a very successful approach to solving crystal structures of coiled-coil proteins by the program *AMPLE*, created by these authors (Bibby *et al.*, 2012 ▶). The method is based on *ab initio* (theoretical) creation by *ROSETTA* of a large number of potential models of protein chains (called decoys), selection of several ensembles of most similar fragments of them, and using these ensembles in automatic MR searches with programs *MrBUMP* (Keegan & Winn, 2008 ▶) and *Phaser* (Storoni *et al.*, 2004 ▶). The results of MR searches are not interpreted at this stage, but are submitted for further phasing by the program *SHELXE* (Sheldrick, 2008 ▶) and automatic model rebuilding by *ARP*/*wARP* (Perrakis *et al.*, 2001 ▶) or *Buccaneer* (Cowtan, 2006 ▶). The whole, highly automated, *AMPLE* pipeline is therefore a very illustrative example that, paraphrasing the expression of Newton, science is advanced by researchers ‘standing on the shoulders of colleagues’ (Newton, 1676 ▶).

The performance of the method was tested on a set of almost a hundred diverse coiled-coil structures selected from the PDB. About 80% of them were successfully solved without human intervention, including protein structures up to 250 residues long and cases when crystals diffracted only to about 3 Å resolution, as well as for some complexes where coiled-coil fragments constitute only part of the whole structure. This approach is therefore significantly more successful than the traditional way, which utilizes single search models selected from the PDB. Owing to the high level of automation, the whole process is relatively fast and does not involve much human intervention. *AMPLE* is available as a part of the *CCP*4 suite of programs.

The paper by Rämisch *et al.* (2015 ▶) in *Acta Cryst. D* presents another efficient pipeline combining *de novo* structure prediction, MR search and automated model building, called *CCsolve*. It uses the *Fold-and-Dock* protocol of *ROSETTA* to create optimized search models from the known protein sequence and its oligomeric state. The sequence is initially analyzed by the secondary-structure prediction program *PSIPRED* (Jones, 1999 ▶), and the unstructured parts of the chain (at termini) are removed. The 20 best *ab initio* models are then further modified by changing a number of side chains to alanine and such ‘quasi-polyalanine’ models in the form of single helices and as predicted oligomers are submitted to *Phaser*. Several best MR solutions are then refined by *phenix.refine*, and automatically rebuilt by *PHENIX* in the *AutoBuild* mode (Terwilliger *et al.*, 2008 ▶).

The *CCsolve* approach was benchmarked against 24 coiled-coil structures selected from the PDB, and 22 of them were successfully solved by this approach. The program is freely available from the author’s web page.

*AMPLE* and *CCsolve* constitute a very valuable extension to the tools available to structural biologists, significantly enhancing the ‘solvability’ of crystal structures of an important class of proteins, those built from long α-helices that used to be notoriously difficult to solve.
